# Nosocomial pneumonia in critically ill patients

**DOI:** 10.4103/0970-2113.68321

**Published:** 2010

**Authors:** Girish L. Dandagi

**Affiliations:** *Department of Pulmonary Medicine, KLE’S Jawaharlal Nehru Medical College and Research Centre, Belgaum, India*

**Keywords:** Hospital acquired pneumonia, nosocomial pneumonia, ventilator associated pneumonia

## Abstract

The care of critically ill patients in the intensive care unit (ICU) is a primary component of modern medicine. ICUs create potential for recovery in patients who otherwise may not have survived. However, they may suffer from problems associated with of nosocomial infections. Nosocomial infections are those which manifest in patients 48 hours after admission to hospital. Nosocomial infections are directly related to diagnostic, interventional or therapeutic procedures a patient undergoes in hospital, and are also influenced by the bacteriological flora prevailing within a particular unit or hospital. Urinary tract infections are the most frequent nosocomial infection, accounting for more than 40% of all nosocomial infections. Critical care units increasingly use high technology medicine for patient care, hemodynamic monitoring, ventilator support, hemodialysis, parenteral nutrition, and a large battery of powerful drugs, particularly antibiotics to counter infection. It is indeed a paradox that the use of high-tech medicine has brought in its wake the dangerous and all too frequent complication of nosocomial infections

## INTRODUCTION

Nosocomial pneumonia (NP) is defined as an infection of the lung parenchyma that was neither present nor incubating at the time of hospital admission and which develops after 48 hours of hospital admission.[1] Data from the National Nosocomial Infections Surveillance system (NNIS) of the United States suggests nosocomial pneumonia as the second most common nosocomial infection in intensive care units.[1] Additionally pneumonia is associated with the greatest mortality among nosocomial infections and with considerably increased costs of care.[1] The widespread use of tracheal intubation and mechanical ventilation to support the critically ill patients further increases in patients who are already high risk for development of NP.[1] Despite advances in the diagnosis and treatment, our understanding of the NP remains incomplete. The incidence of NP in the ICU ranges from 9 –24% with variation relating to the intensive care and differences in the definitions and diagnostic techniques used.[2]

In India[3] incidence of postoperative infections in various hospitals varies from 10-25%.

Despite availability of newer antimicrobials the treatment of NP has proved to be difficult. The clinical presentation and organisms causing NP are different in different organizations. Hence there is every need for early diagnosis and management of these patients to decrease morbidity and mortality.

The American Thoracic Society (ATS) con-sensus statement[4] suggests the cat-egorization of NP as early-onset NP, which occurs within four days after hospital admission and late-onset NP, which occurs after five days of hospital admission. Most cases of NP in the ICU occur in patients who are tracheally intubated and receiving mechanical ventilation.[4]

Ventilator associated pneumonia (VAP) is defined as pneumonia occurring after 48 hours of endotracheal intubation and initiation of mechanical ventilation[5] Ventilator associated pneumonia (VAP), cat-egorized as early-onset VAP, which occurs within four days of endotracheal intubation whereas late-onset VAP occurs after four days of endotracheal intubation. This classification also helps in predicting the implicated pathogens and guides us in the initial empiric therapy with antibiotics.[6]

The accuracy of epidemiologic data of NP and VAP has been in question because of the difficulties in defining a “gold standard” for diagnosis.[7] The incidence of NP varies from hospital to hospital.[7] The incidence of NP in the ICUs ranges from 9 to 24 % with variation relating to care presented in the ICUs and differences in the diagnostic techniques used.[7] In an Indian study NP was found to have a 9% incidence using radiological and microbiological criteria.[7] A large-scale, prevalence study nosocomial pneumonia arising in the ICU was performed as a part of the European prevalence of infection in Intensive Care (EPIC) study. Among a total of more than 10000 patients in 1417 ICUs across Europe the overall NP prevalence was 9.6%.[8] In this study, logistic regression analysis identified mechanical ventilation as one of the several risk factors for ICU-acquired infections. Using a protected specimen brush (PSB) during fiberoptic bronchoscopy to diagnose pneumonia in ventilated patients, Fagon *et al*,[9] reported a NP incidence rate of 9%. An incidence of 16.6% was reported at a later period using bronchoscopic protected specimen brush (PSB) and bronchoalveolar lavage (BAL) with quantitative culture techniques.[10] The wide differences in incidence rates is due to the difference in the number of patients in each study, the hospital setting, the diagnostic criteria used to confirm pneumonia, and the mean duration of mechanical ventilation in the study population.[7]

Early onset VAP is caused by *Streptococcus pneumonia, H influenza, Moraxella catarrhalis* and methicillin-sensitive *Staphylococcus aureus* (MSSA). Late onset VAP is caused commonly by *Pseudomonas aeruginosa, Acinetobacter, Enterobacter* species and methicillin resistant *staphylococcus aureus* (MRSA).[11] VAP is frequently polymicrobial and gram negative bacilli are the predominant organism’s isolated.[12]

The risk of acquiring pneumonia appears to increase with the duration of mechanical ventilation in one study it was found to be 7% at 10 days and 19% at 20 days.[13] In that study the incremental risk of pneumonia was virtually constant with a mean rate of around 1% per day of ventilation. In another large series,[14] the cumulative risk of developing VAP was seen to be maximum up to day 5, with the rate declining thereafter. The risk per day was estimated to be 3% on Day 5.2% on Day 10 and 1% on Day 15. The incidence is higher in surgical than in medical ICUs.[15] In cardiothoracic ICUs, the incidence is about 22%; in other surgical ICUs, it is around 14%; and in medical ICUs, it is approximately 9%.

There is another study[16] which includes 201 patients (1285 patient days) admitted over a period of one-and-a-half years. A total of 77 infections were identified in 67 patients (33.5%).

The infections included pneumonia (23%), sepsis of unknown origin (10.5%), bacteremia (7.5%), and urinary tract infections (1.5%). The most commonly identified organisms were the clostridium difficile colitis (1%). The most commonly identified organisms were Acinobacter species (34.8%), *Pseudomonas aeruginosa* (23.9%) and *Escherichia coli* (15.2%).

Trivedi *et al*,[17] reported an incidence of 9.38% of NP and 38% had ventilator associated pneumonia. Commonest isolates were pseudomonas (55%), *Acinetobacter* (20%), *Staph. aureus* (14.5%) and *Klebsiella* (75%). Total mortality was 21.3%.[7] VAP is associated with crude mortality rate of 20 –70%. Mortality as a result of VAP is especially high when it is caused by multi-drug resistant organisms like pseudomonas or *Acinetobacter* species. Kollef reported that overall mortalities in patients with VAP were 37.5% as compared with 8.5% in patients without pneumonia.[15] NP has been identified as an important prognostic factor in different groups of critically ill patients.

The mortality rates in patients with NP are higher than in patients without NP, but whether this reflects a direct cause – effect relationship is uncertain.[2] It is perhaps, more a reflection that patients who develop nosocomial infection are already in a high risk group of critically ill patients with higher mortality rates than the rest of the population.[2] Currently the exact role of nosocomial infection themselves in worsening the prognosis of ICU patients is difficult to assess, as such patients are critically ill and thus their clinical status is severe enough to require ICU care and potentially to cause death. Thus, although rates of NP and mortality are high, assessment of responsibility of several other risk factors that confound this relationship is difficult.[10] Therefore it is difficult to establish whether the patients would have survived if the pneumonia did not occur. Several factors have been associated with a greater risk of mortality and the most common factors are *Pseudomonas aeruginosa* as a pathogen, severity of underlying illness, inappropriate antibiotic therapy and advanced age. The etiologic agents that cause ventilator associated pneumonia are distinct from that of community acquired pneumonia.

The common pathogens[18] associated with NP are listed in Table 1.

**Table 1:** Common pathogens associated with nosocomial pneumonia and ventilator assiciated pneumonia

Pathogens	Frequency%
I. Common pathogens associated with NP	
A] Early onset bacteria pneumonia	
*Streptococcus pneumoniae*	5-20
*H. influenzae*	<5-15
B] Late onset bacterial pneumonia:	
1) Aerobic Gram-negative bacilli:	20-60
* P. aeruginosa*	
* Enterbecter spp*	
𠀺*Acinetobacter spp*	
* K. pneumoniae*	
* S. marcescens*	
*E. coli*	
2) Gram-positive cocci	20-40
*S. aureus*	
C] Early and late onset pneumonia:	
1) Anaerobic bacteria	0-35
2) *L. pneumophilia*	0-10
3) *P. carinii*	<1
4) *M. tuberculosis*	<1
5) Viruses:	
Influenza A and B	<1
Respiratory synctial virus	<1
6) Fungal:	
*Aspergillosis*	<1
*Candida spp*	<1
II. Common pathogens currently associated with VAP	
A] Early onset VAP	
1) Gram-positive cocci	
*Streptococcus pneumoniae*	
Methicilin sensitive *Staphylococcus aureus (MSSA)*	
2) Gram negative bacilli	
*E. coli*	
*Klebsiella* species (spp)	
*Serratia marcescens*	
B] Late onset VAP:	
1) Gram-positive cocci	
Methicilin resistant *Staphylococcus aureus* (MRSA)	
2) Gram negative bacilli	
*Enterobacteriaceae* species	
*Pseudomonas aeruginosa*	
*Acinetobacter baumanii*	

The predictors of VAP also depended on the type of patient and were different for different types of illness. The incidence of NP may be also age-dependent, with about five NPs per 1000 inpatients aged under 35 and 15 NPs per 1000 in-patients aged above 65.[19–21]

## PATHOGENESIS

For any infection to occur there must be interplay of three factors - impaired host defense, access of pathogenic bacteria in sufficient numbers to the lower respiratory tract and the virulence of the organism.[22] The organism may gain access into the lungs by one of several routes i.e.; micro aspiration of oropharyngeal secretions, aspiration of gastric contents, inhalation, hematogenous spread, direct inoculation and exogenous penetration (e.g. pleural space). Of these, micro-aspiration is the most common.

### Various routes of bacterial entry are as follows

Micro-aspiration of oropharyngeal secretions colonized with pathogenic bacteria, Aspiration of esophageal / gastric contents, Inhalation of an infected aerosol, Hematogenous spread of infection from a distant site of infection, exogenous penetration from an infected site (i.e. pleural space), and direct inoculation into the airway of incubated patients from ICU personnel.[4]

### Risk factors for development of nosocomial pneumonia

Mechanical ventilation, particularly prolonged coma or reduced conscious level, supine positioning, aspiration, pre-existing disease – e.g.: chronic obstructive pulmonary disease (COPD), admitting diagnosis – trauma, burns, prolonged ICU stay, use of PEEP during mechanical ventilation, high disease severity (APACHE II score), multiple organ dysfunction, older age, prior administration of antibiotics, malnutrition, use of nasogastric tube, use of paralytic agents, administration of antacids, male gender, enteral feeding and immunosupression.[2]

**Figure 1:**
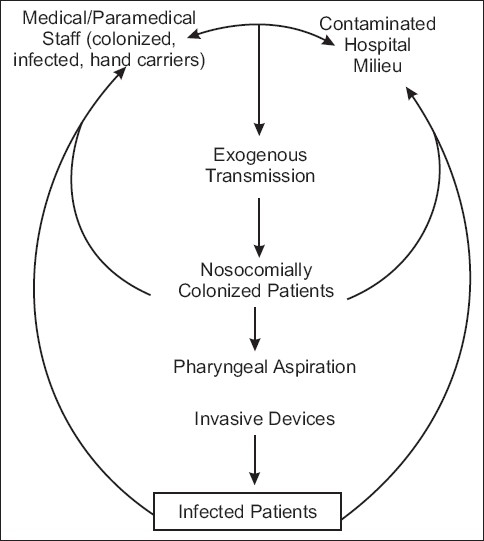
Cycle of transmission of nosocomial pneumonia. (Courtesy: Faroch Erach udwadia Editor, Textbook of Priciples of Critical Care, Chapter 13.2, NSOCO. Mail Infections published by Oxford University Press, New Delhi, 2nd Edition, 2005 P 384.)

The intensive critical care unit (ICCU) is a closed milieu, constantly full of patients with life-threatening illnesses. It has a constant staff of nurses and doctors who over prolonged periods of time are exposed to and are in contact with an environment contaminated by antibiotic-resistant pathogens. In this closed milieu [Figure 1] the major reservoir of nosocomial organisms is the infected or colonized patient. Most bacteria, many viruses, and possibly even fungi are spread primarily via the hands of the medical, paramedical and nursing staff.

The risk factors include patient related risk factors, infection control related factors and intervention related factors.[4]

Defining severe hospital acquired pneumonia[4]

1. Admission to the ICU
2. Respiratory failure, defined as the need for mechanical ventilation or the need for> 35% oxygen to maintain an arterial oxygen saturation > 90%
3. Rapid radiographic progression, multilobar pneumonia, or cavitation of a lung infiltrate
4. Evidence of severe sepsis with hypotension and/or end-organ dysfunction:
  Shock (systolic blood pressure < 90 mm Hg, or diastolic blood pressure < 60 mm Hg), Requirement for vasopressors for more than four hours, urine output < 20 ml/h or total urine output < 80 ml in 4 h (unless another explanation is available), Acute renal failure requiring dialysis.

### Clinical diagnostic criteria of NP and VAP

Clinical suspicion of pneumonia with a new or progressive chest radiographic infiltrates after 48 hours of admission or after 48 hours of patients on mechanical ventilation and one of the following:

1. Fever > 38.3 c
2. Leukocytosis > 12000/cmm or Leucopenia < 4000/cmm
3. Purulent respiratory secretions with gram stain demonstration of bacteria and polymorphs.
4. Cultures with growth > 10 ^6^ colony forming units (cfu)/mL.

Despite numerous high quality studies addressing different diagnostic strategies in the setting of suspected VAP, no single approach meets anything close to majority approval. The optimal diagnostic and management strategy for VAP remains controversial. Unlike community-acquired pneumonia identification of an infiltrate in the lungs as pneumonia in the ICU setting is far more complex as there are several pitfalls in the diagnosis.

Methods to obtain culture material from the lower respiratory tracts:-

- Non invasive (minimally invasive)
  Endotracheal aspirate – (standard)
  Simplest method
- Non bronchoscopic techniques
  1. Plugged telescoping catheter (PTC),
  2. Protected bronchoalveolar mini-lavage (mini-PBAL), and
  3. “Blind” Protected specimen brushing.
- Invasive; Bronchoscopic techniques –Protected specimen brushing (PSB) and BAL (Bronchoalveolar Lavage), Open lung biopsy

## TREATMENT

The current treatment of NP relies on appropriate antimicrobial therapy and inadequate antibiotic therapy is associated with increased mortality rates. However, adequate treatment can be a challenge due to the range of organisms’ encountered the high incidence of resistant organisms and frequency of polymicrobial nature of nosocomial infections. Several specific issues relating to antibiotic selection were considered, including the expected efficacy of an appropriate therapeutic choice. Specific pharmacologic features of antimicrobial agents should also be considered, including cost. The penetration of antibiotics to the site of infection is important, but it remains unclear whether concentrations in bronchial secretions or in epithelial lining fluid are most relevant for predicting efficacy. Some agents penetrate into respiratory secretions better than others. American Thoracic Society[3] suggested the following treatment plan. Aminoglycosides have relatively poor penetration, while fluoroquinolones can achieve better concentration in bronchial secretions. Agents such as the aminoglycosides and quinolones are bactericidal in a concentration dependent fashion. In addition these agents have a prolonged postantibiotic effect (PAE), allowing them to suppress bacterial growth even after their concentrations are below target level. Other agents such as vancomycin and the beta-lactams are also bactericidal but act in a time-dependent rather than in a concentration-dependent fashion, do not possess significant postantibiotic effect against gram negative bacilli, is seen with beta-lactam antibiotics (penicillins, cephalosporins, aztreonam). One exception is the beta-lactam carbapenems antibiotics such as imipenem. Generally speaking, empirical therapy should be commenced once NP is suspected and altered as microbiologic data become available. The ATS (American Thoracic Society)[3] has produced a consensus statement suggesting various treatment strategies, based on the division of patients into groups according to the severity of their disease and the presence of associated risk factors.

## PREVENTION

The two important processes involved in the pathogenesis of hospital acquired pneumonia are:

1. Bacterial colonization of the aerodigestive tract and
2. Aspiration of the contaminated secretions into the lower airway.

Therefore, preventive strategies for hospital acquired pneumonia are directed at reducing the bacterial burden colonizing the aerodigestive tract, and decreasing the occurrence of aspiration

## SOME PREVENTIVE STRATEGIES

1. Thorough hands washing is the simplest and most effective means of limiting spread of infection but is frequently inadequate or/ not performed at all.
2. Heat and moisture exchanges may decrease the incidence of NP. However, not all studies confirm this.
3. Noninvasive ventilation has been associated with reduced rates of infection and should be considered in appropriate patients.
4. Nursing patients in the supine position may increase the risks of pulmonary aspiration of gastric contents. Several studies have confirmed reduced rates of NP in patients nursed in semirecumbent rather than supine and this should be encouraged although it is not always practically possible.
5. Avoiding excessive sedation – sedation should be titrated to minimal level required to keep patient comfortable.
6. Several authors have suggested an increased incidence of pneumonia with antacids and H _2_ blockers. Routine use of antacid strategies should be avoided.
7. Selective digestive decontamination (SDD): Consists of non absorbable tropical antibiotics (Polymycin, tobramycin and amphotericin B) plus the use of systemic antibiotics (cefotaxime). Many studies have shown that SDD reduces NP. However, concern has been raised about risks of encouraging antimicrobial resistance and this has not gained wide acceptance.
8. Kinetic beds and continuous subglottic suctioning of secretions that pool above endotracheal different cuff both are expensive and not widely used.
9. Simple techniques such as had washing, placing the patient in semirecumbent position and avoiding excess sedation must become a routine part of ICU care.

## CONCLUSION

Early diagnosis and prompt initiation of antibiotic therapy can help reduce the increased medical and economic burdens associated with NP.
